# Comparison of Faecal Anaerobic Fungi of Cows and Goats Offered Similar Diet

**DOI:** 10.1002/vms3.71291

**Published:** 2026-10-30

**Authors:** Tiziana Maria Mahayri, Alessia Serra, Janet Luthar, Radka Roubalová, Giuseppe Moniello, Silvana Mattiello, Stefania Celozzi, Jakub Mrázek, Kateřina Olša Fliegerová

**Affiliations:** ^1^ Laboratory of Anaerobic Microbiology Institute of Animal Physiology and Genetics, Czech Academy of Science Prague Czech Republic; ^2^ Department of Veterinary Medicine University of Sassari Sassari Italy; ^3^ Laboratory of Cellular and Molecular Immunology Institute of Microbiology of the Czech Academy of Sciences Prague Czech Republic; ^4^ Department of Agricultural and Environmental Sciences—Production, Landscape, Agroenergy University of Milan Milan Italy

**Keywords:** anaerobic fungi, cow, goat, microbiota, ruminants

## Abstract

**Background:**

Anaerobic fungi (AF) are key contributors to fibre degradation in ruminants, yet their host‐associated patterns remain poorly understood. The aim of this study was to compare the diversity and composition of AF in faeces of goats and cows offered similar diet and to evaluate the influence of animal species on the gut mycobiome.

**Methods:**

A total of 15 female goats (Blond Adamellan) and 16 female cows (Brown Swiss) kept on an organic farm were fed pasture and hay. The fungal structure in faeces was examined by high‐throughput sequencing using the D2 LSU region.

**Results:**

Alpha diversity measurements revealed significantly lower species richness and evenness in goats compared to cows. Beta diversity analysis showed statistical dissimilarities and distinct community structures between the two ruminant species confirming host‐specific clustering. Taxonomic composition differed significantly, cow samples were dominated by Caecomycetaceae and Neocallimastigaceae, particularly the genera *Orpinomyces*, *Caecomyces* and *Cyllamyces*. Goat samples were dominated by *Anaeromyces*, *Caecomyces* and according to relative abundance they formed two distinct clusters with highly similar fungal profile. Differential abundance analysis further highlighted the enrichment of *Anaeromyces*, unclassified genus within Caecomycetaceae, *Feramyces* and *Pecoramyces* in goats, whereas *Caecomyces* and *Piromyces* were more abundant in cows.

**Conclusion:**

These findings underscore the strong influence of host phylogeny on the faecal anaerobic fungal community.

## Graphical abstract

Faecal anaerobic fungal communities differed between cows and goats fed similar diet: Goats showed lower diversity and enrichment of *Anaeromyces*, *Feramyces* and *Pecoramyces*, whereas cows were enriched in *Caecomyces* and *Piromyces*. These findings highlight the influence of host species on the gut mycobiome.

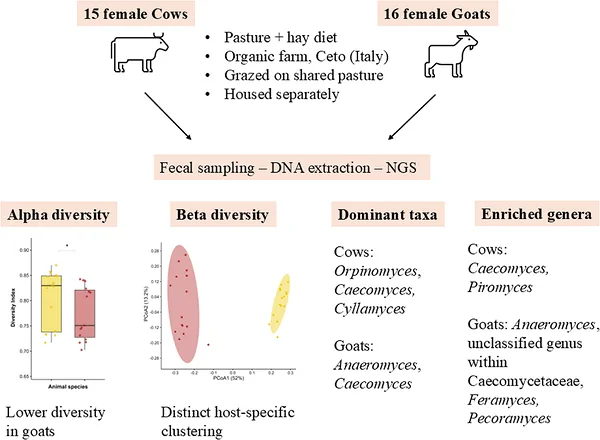

## 1 Introduction

Ruminants are extremely important livestock animals due to their ability to convert lignocellulosic feed, often of low quality, into animal‐based products such as milk and meat for human consumption (Pulina et al. 2017; Wang et al. 2024). This unique capability is largely attributed to the complex microbial communities residing in the rumen (Huws et al. 2018). The ruminal ecosystem comprises several groups of microorganisms, including bacteria, protozoa, archaea, and fungi, which are responsible for the degradation of biomass to volatile fatty acids (VFAs) which serve as the host's primary energy source (Hess et al. 2020; Wang et al. 2020). Research has focused on bacteria, which represent the most abundant component of the rumen microbiome (Shanks et al. 2011; Jewell et al. 2015; Liu et al. 2016; Noel et al. 2019; Mahayri et al. 2026). Archaea have received increased attention due to their crucial role in methane emission (Beauchemin et al. 2020; Khairunisa et al. 2023; Malik et al. 2023), and rumen ciliates have also been studied for their involvement in fibre degradation and proteolysis (Beauchemin et al. 2020). In contrast, anaerobic fungi (AF) are the least studied components of herbivore microbiome.

However, studies in the last decade have shown that AF play an absolutely crucial role in the decomposition of biomass and their contribution to the degradation of dietary fibre is significant (Gruninger et al. 2014; Choudhury et al. 2015; Hess et al. 2020; Kovács et al. 2025). Transcriptomic and proteomic studies revealed a large array of biomass‐degrading enzymes belonging to the broad range of glycoside hydrolase (GH) families, which are able to decompose efficiently recalcitrant (hemi)cellulose structures of crude, untreated plant biomass (Couger et al. 2015; Henske et al. 2017; Hagen et al. 2021). Moreover, these enzymes can be organized in cellulosomes and thus can work synergistically, which makes AF the most active plant‐degrading microbes in nature (Haitjema et al. 2017).

Recent research has made significant progress in understanding AF taxonomy. Currently, 14 genera of AF have been taxonomically classified in the phylum Neocallimastigomycota, class Neocallimastigomycetes, order Neocallimastigales and families Neocallimastigaceae, Piromycetaceae, Caecomycetaceae and Anaeromycetaceae. However, six genera have been designated as ‘genera incertae sedis’ in the order Neocallimastigales due to the deep branching or uncertain position (Hanafy et al. 2023). The position of the two most recently described fungi, *Testudinimyces* and *Astrotestudinimyces* (Pratt et al. 2023), also remains unresolved. Thus, the AF taxonomy remains problematic, and diversification will be further complicated by newly predicted genera (Meili et al. 2023).

Meili et al. (2023) conducted an extensive analysis of AF community structure, examining 661 faecal samples collected from 34 mammalian species across 6 continents and greatly expanded known AF diversity by identifying 56 novel genera. Moreover, they observed that host phylogenetic affiliation is the main factor shaping the community, whereas other factors, such as domestication status and biogeography, play a less influential role.

Diet certainly also plays an important role in shaping AF diversity, but the impact of feed on AF community structure appears less decisive than for bacteria. Henderson et al. (2015) reported that differences in rumen bacterial community composition were predominantly determined by diet, with host affiliation being less influential. However, the influence of diet and host species requires further investigation. Meili et al. (2023) noted that the dataset used in their study was not optimal, as a statistically reliable number of AF‐sequenced replicates came from domesticated animal species fed a very similar diet.

To contribute to the clarification of this issue, we used samples from our previous study, in which we compared the faecal bacterial composition of cows and goats grazing on the same pasture. We showed that host species affected bacterial evenness, but not diversity (Mahayri et al. 2022).

In the present study, we examined and compared the diversity and composition of AF in the faeces of cows and goats grazing on an organic farm. Both groups of animals were housed together, fed the same high‐fibre diet (pasture and hay) and kept in the same location (Ceto, Italy).

Using tailed‐amplicon sequencing with a specific primer pair targeting the Neocallimastigomycetes rDNA large subunit (LSU) D2 region (D2 LSU), we compared the fungal diversity and taxonomic composition between the two ruminant species. On the basis of literature data, we hypothesize that the AF population in samples from two ruminant species fed similar diet will differ, as host phylogenetic affiliation shapes the AF community rather than other factors.

## 2 Materials and Methods

### 2.1 Animals and Sample Collection

Samples were collected from 31 animals, 15 female Blond Adamellan goats and 16 female Brown Swiss cows, kept on a private organic farm in Ceto, Lombardy, Italy (latitude: 46°22′00″; longitude: 10°21′09″), as previously described (Mahayri et al. 2022). Briefly, the animals grazed freely in separate pastures during the day (7:30–17:00). The botanical composition of the sward includes many different species and varies mainly depending on the altitude (from 400 to 2000 m a.s.l.), ranging from pastures dominated by *Festuca* spp. to pastures where *Carex* spp. and *Sesleria* spp. are the main botanical species. For the remainder of the time, cows and goats were kept in separate housing systems on the same farm—goats in freestalls, cows in tie stalls. The animals were offered mixed hay (50% polyphyte hay/50% alfalfa hay) ad libitum three times daily and had free access to water both indoors and outdoors.

Both ruminant species underwent external antiparasitic treatment with eprinomectin (0.5 mg/kg body weight) simultaneously 45 days before sampling. The characteristics of the animals are given in Table S1.

Fresh faecal samples were collected outdoors immediately after defecation and processed as previously reported by Mahayri et al. (2022). The freeze‐dried samples were sent to the Institute of Animal Physiology and Genetics of the Czech Academy of Sciences in Prague, Czech Republic, for further analysis.

### 2.2 DNA Extraction

DNA extractions from freeze‐dried faecal samples were performed using DNeasy Plant Pro Kit (Qiagen, Hilden, Germany) according to the manufacturer's instructions. As a preliminary step, the samples were crushed in liquid nitrogen using a mortar and pestle to break up the fungal cell walls containing chitin. A powdered portion equivalent to 300 mg of wet weight was used for nucleic acid extraction. The concentration and purity of the extracted DNA (260/280 ratio) were assessed using a NanoDrop 2000c UV–Vis spectrophotometer (Thermo Scientific, Wilmington, DE, USA). The DNA extracts were stored at −20°C until further analysis.

### 2.3 PCR Amplification and Purification

The DNA extracted from each sample was diluted 10‐fold in nuclease‐free H_2_O and 1 µL (approximately 20 ng/µL) was used as the template for amplification of the rDNA LSU D2 region. PCR reaction was performed using DreamTaq Green PCR Master Mix (Thermo Fisher Scientific) with tailed primers AGF‐LSU‐tail F (5′‐TCG TCG GCA GCG TCA GAT GTG TAT AAG AGA CAG GCG TTT RRC ACC AST GTT GTT‐3′) and AGF‐LSU‐tail R (5′‐GTC TCG TGG GCT CGG AGA TGT GTA TAA GAG ACA GGT CAA CAT CCT AAG YGT AGG TA‐3′) (Young et al. 2022) under the following cycling conditions: an initial denaturation for 3 min at 95°C, followed by 35 cycles of 20 s at 95°C, 30 s at 58°C, 1 min at 72°C, and a final elongation step at 72°C for 10 min. The length and quality of the amplicons were assessed by agarose gel electrophoresis (1.5%), and the PCR products were purified using the Monarch PCR and DNA Cleanup Kit (New England BioLabs, Ipswich, MA, USA). Concentrations were measured with Qubit 2.0 Fluorometer (Thermo Scientific, Wilmington, DE, USA) using the Qubit dsDNA HS (high sensitivity) Assay Kit. Samples were then diluted to 5 ng/µL, as required by SeqMe company (Seqme.eu, Dobříš, Czech Republic) and sent for Illumina sequencing.

### 2.4 Library Preparation and High‐Throughput Sequencing

Library preparation was performed by SeqMe company (Seqme.eu, Dobříš, Czech Republic) using a tailed amplicon library approach with Nextera technology. Sample‐specific indexes were incorporated through index PCR using the Nextera XT system. Library quality was assessed with an Agilent Bioanalyser 2100 (Agilent Technologies, Santa Clara, CA, USA) and an HS DNA Kit. Quantification was performed using an Invitrogen Collibri Library Quantification Kit (Thermo Scientific, Wilmington, DE, USA) and Quant‐iT 1X dsDNA HS Assay (Thermo Scientific, Wilmington, DE, USA). The final libraries were pooled equimolarly and sequenced on an Illumina NovaSeq 6000 system.

### 2.5 Bioinformatics and Statistical Analysis

The sequences were exported in FASTQ format and analysed using Qiime2 version 2020.2 software (Bolyen et al. 2019). Quality filtering, trimming, denoising and chimaeras removal were conducted using the DADA2 (Callahan et al. 2016). Rarefaction was performed to ensure sampling depth; the dataset was subsampled to a minimum of 18,000 reads per sample. Rarefaction curves reached a plateau revealing thus sufficient coverage of fungal diversity (Figure S1). The number of raw reads and filtered high‐quality reads for each sample is reported in Table S2. Sequences were then clustered into amplicon sequence variants (ASVs) using VSEARCH and the taxonomic assignment was performed using a BLAST search against the AF_LSU database version 2.0, available on the website of the Anaerobic Fungi Network (www.anaerobicfungi.org). The anaerobic fungal diversity was evaluated using alpha diversity indices Shannon Entropy, Chao1, Faith's Phylogenetic Diversity and Pielou's Evenness Index. Group comparisons were evaluated using the Kruskal–Wallis *H* test and visualized using tidyverse, qiime2R, ggplot2, and ggpubr packages in RStudio (version 4.4.3). ASVs were aligned with MAFFT, and the alignment was mask‐filtered prior to tree construction with FastTree. The resulting rooted tree was used for Faith's PD and UniFrac distance calculations. Beta diversity was assessed using weighted UniFrac distance matrix. The principal coordinate analysis (PCoA) was used to visualize the community clustering, and the results were plotted using ape, qiime2R, ggplot2 and ggpubr packages in RStudio. Permutational multivariate analysis of variance (PERMANOVA) was performed using 999 permutations to assess the statistical difference between the groups. In addition, the PERMDISP test was performed to evaluate the homogeneity of group dispersions. Differential abundance analysis was carried out using the DESeq2 package in R. Prior to analysis, low‐coverage taxa with less than 10 raw counts across all samples were removed. Raw data were normalized using DESeq2 default median‐of‐ratios method. To adjust for high variance in low‐abundance taxa, log2 fold changes (log2 FC) were estimated using effect size shrinkage. Estimated *p* values were corrected for multiple testing using the Benjamini‐Hochberg correction. Taxa were considered differentially abundant at an adjusted *p* value <0.05.

## 3 Results

### 3.1 Alpha and Beta Diversity

The anaerobic fungal community structure in the faeces of cows and goats offered the similar diet was qualitatively and quantitatively analysed for species richness, evenness and phylogenetic biodiversity. Significant differences in Shannon Entropy (*p* = 0.000002), Chao1 index (*p* = 0.000002), Pielou's evenness index (*p* = 0.04) and Faith's phylogenetic distance (*p* = 0.000002) were observed between the two studied groups, as shown in Figure 1 and summarized in Table S3. The analysis revealed a lower diversity in the samples of goats. The differences in the fungal community related to host species were disclosed also by beta diversity analysis. A PCoA based on the weighted UniFrac distance matrix shows spatial separation of the cow and goat samples (Figure 2). Statistical analysis revealed a significant difference between the groups (PERMANOVA *p* = 0.001, *R* ^2^ = 0.438). This model indicates that 43.8% of the variation in anaerobic fungal community can be attributed to host identity, which represents a substantial effect size. The PERMDISP test confirmed homogeneity of dispersion across groups (*p* = 0.22), supporting that the observed differences were driven by compositional shifts rather than intergroup variability.

**FIGURE 1:**
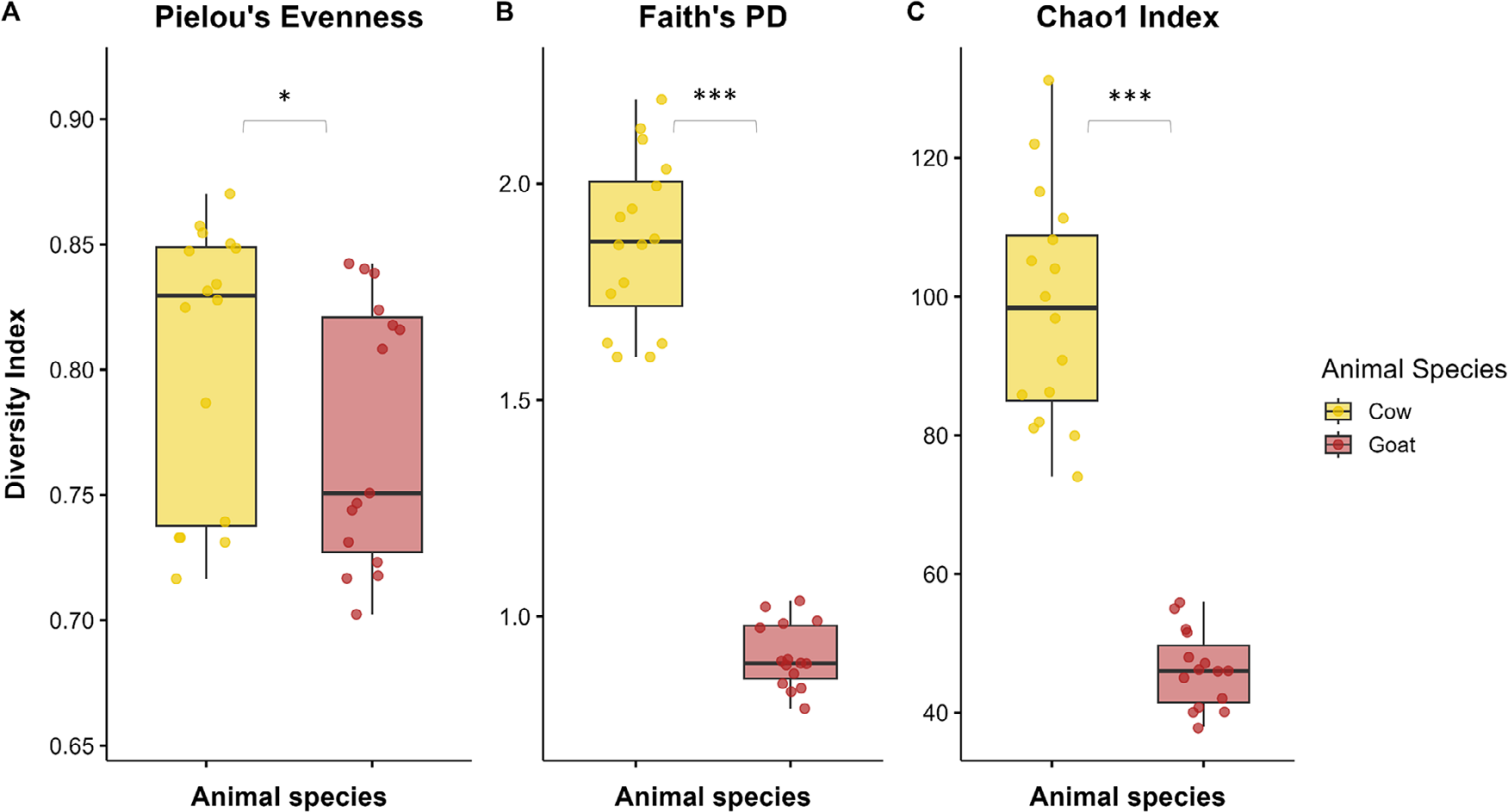
Comparison of alpha diversity indices for faecal anaerobic fungal community of cows and goats: (A) fungal evenness estimated by Pielou's evenness index, (B) fungal phylogenetic diversity estimated by Faith's PD, and (C) fungal richness estimated by Chao1 index. Statistical significance is indicated as follows: *p* < 0.05 (*)*, p* < 0.001 (***).

**FIGURE 2:**
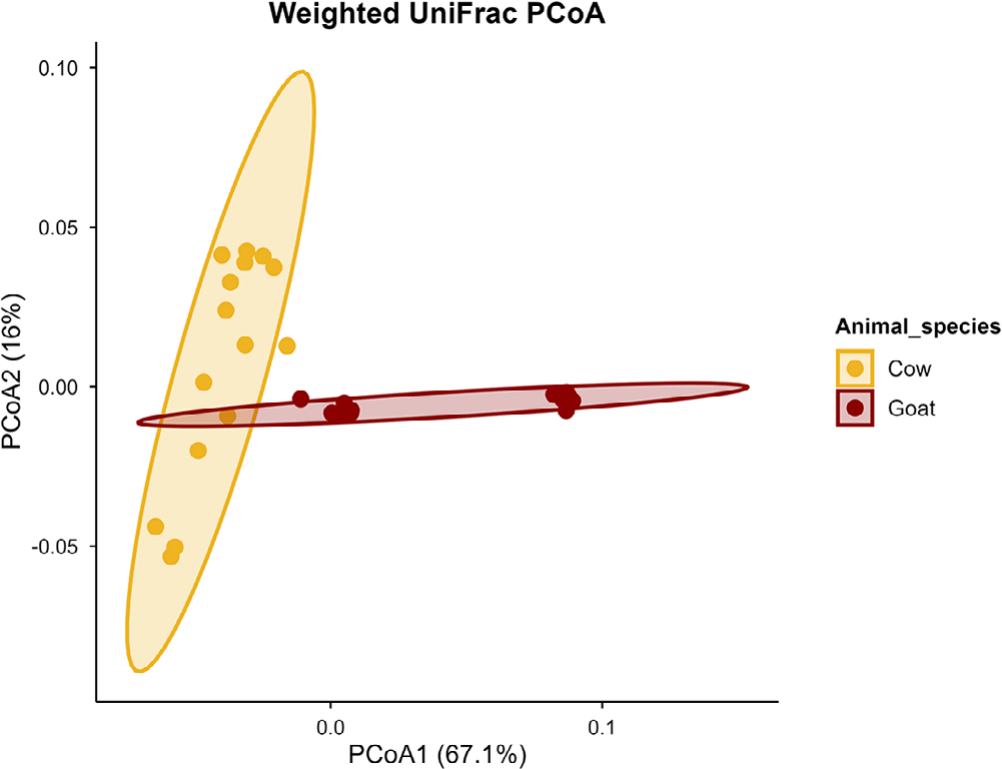
Principal coordinate analysis (PCoA) showing the weighted UniFrac distance matrix between the faecal anaerobic fungal community of cows (yellow colour) and goats (red colour). Each dot represents one sample. The percentage of variation is indicated on the axes (PERMANOVA *p* = 0.001, *R* ^2^ = 0.438).

### 3.2 Taxonomical Composition

Across all samples, the majority of the filtered reads were assigned to Neocallimastigomycota (90.05%–99.9%), indicating high specificity of used primers. Unidentified sequences were detected in very small numbers (∼5%), especially in goat samples. Relative abundance values are reported as mean ± standard deviation (SD). In cows’ samples, the order Neocallimastigales was represented by two dominant families, which were Caecomycetaceae (37.79% ± 9.80%) and Neocallimastigaceae (33.20% ± 13.79%), followed by Anaeromycetaceae (6.75% ± 3.50%) and a taxonomic group with unknown or undefined broader relationships (genera incertae sedis, 11.16% ± 6.3%). In two samples (CN1 and CN19), the Piromycetaceae family formed a significant group (35.33% and 37.27%, respectively). Goats’ samples were predominated by three families, Caecomycetaceae, Neocallimastigaceae and Anaeromycetaceae. Goat samples were evidently divided into two groups. In the first group (*n* = 7), the dominant family was Anaeromycetaceae (46.51% ± 1.14%), followed by Neocallimastigaceae (30.28% ± 1.17%) and Caecomycetaceae (20.39% ± 0.97%). In the second group (*n* = 8), Caecomycetaceae was the most dominant family (60.62% ± 1.72%), followed by Neocallimastigaceae (22.77% ± 1.28%) and Anaeromycetaceae (16.54% ± 2.96%). The abundance of genera incertae sedis and Piromycetaceae was extremely low across all goat samples. At the genus level, altogether 16 genera were identified: 11 cultured genera and 5 uncultured AF. In cows, the most abundant genera were *Orpinomyces* (24.42% ± 11.47%), *Caecomyces* (21.73% ± 7.43%) and *Cyllamyces* (12.54% ± 5.50%), followed by lower abundances of *Anaeromyces* and *Neocallimastix*, only in three samples (CN1, CN19, and CN20) increased abundance of *Piromyces* was detected. These known AF formed together approximately 75% of detected genera. The remaining number was made up of a relatively diverse mixture of nine AF species, among which the genera of groups RH1, SK3 and unclassified genus within family Caecomycetaceae exceeded 3% of relative abundance. In goats, the first group (*n* = 7) was dominated by *Anaeromyces* (46.03% ± 1.12%), followed by *Pecoramyces* (18.72% ± 0.43%), *Orpinomyces* (11.47% ± 0.79%), *Caecomyces* (7% ± 0.56%) and *Cyllamyces* (4.99% ± 0.45%). In the second group (*n* = 8), *Caecomyces* was the dominant genus (42.34% ± 1.22%), followed by *Orpinomyces* (17.09% ± 1.20%), *Cyllamyces* (15.40% ± 0.79%), *Anaeromyces* (14.85% ± 2.79%), and *Pecoramyces* (4.98% ± 0.19%). The composition of the anaerobic fungal community in faeces of cows and goats is illustrated in Figure 3. The relative abundance of fungal taxa at the family and genus levels is listed in Table S4.

**FIGURE 3:**
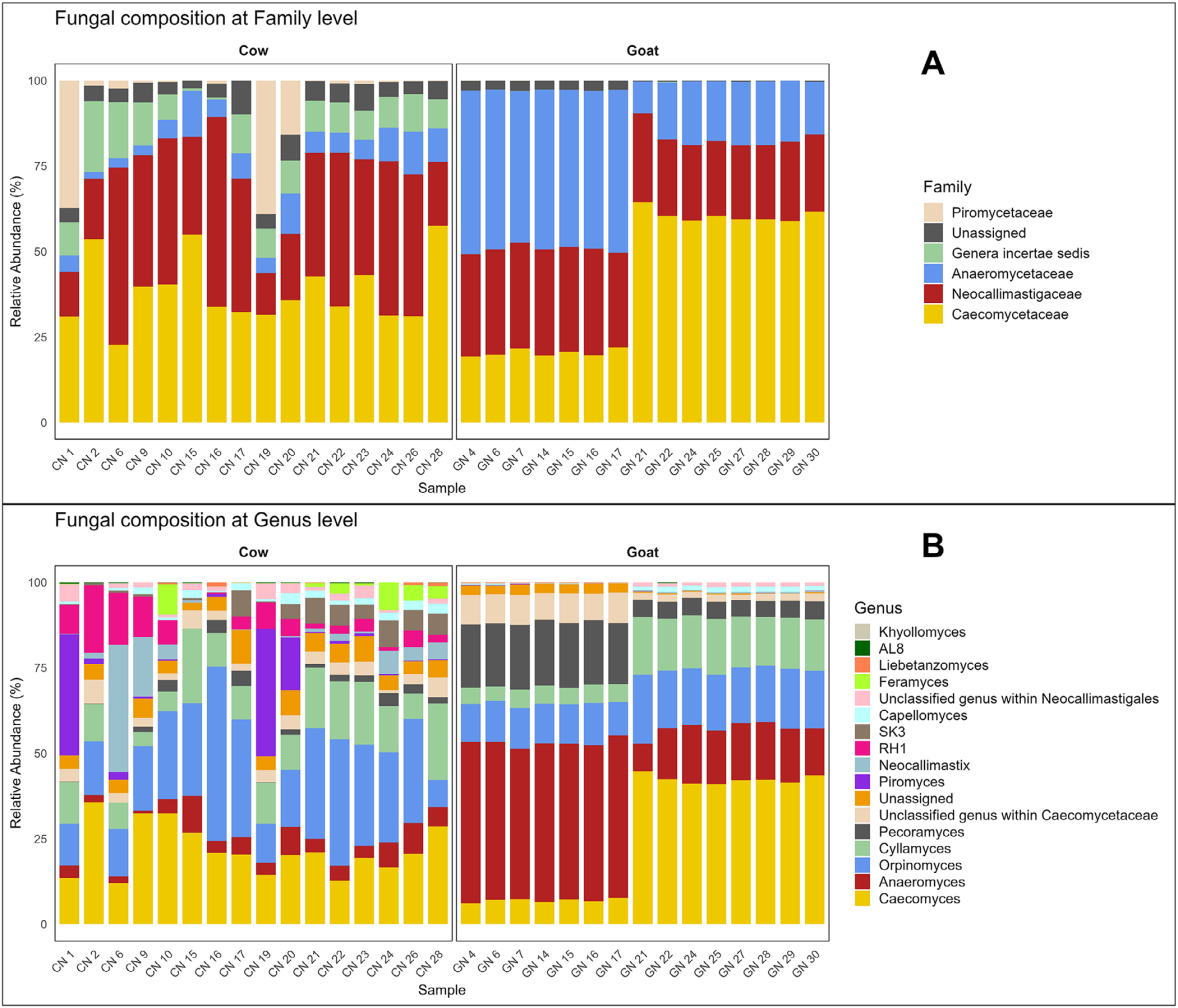
Relative abundance of the faecal anaerobic fungi at family level (A) and genus level (B) in cows and goats offered the similar diet.

### 3.3 Differentially Abundant Taxa

The differential abundance analysis showed that at the family level Anaeromycetaceae was significantly more abundant in goats, whereas Piromycetaceae and Caecomycetaceae were more abundant in cows (Figure 4A, Table 1). At the genus level *Anaeromyces*, *Pecoramyces*, unclassified genus within family Caecomycetaceae, and *Feramyces* were enriched in goats, whereas *Caecomyces* and *Piromyces* were more abundant in cows (Figure 4B, Table 2).

**FIGURE 4:**
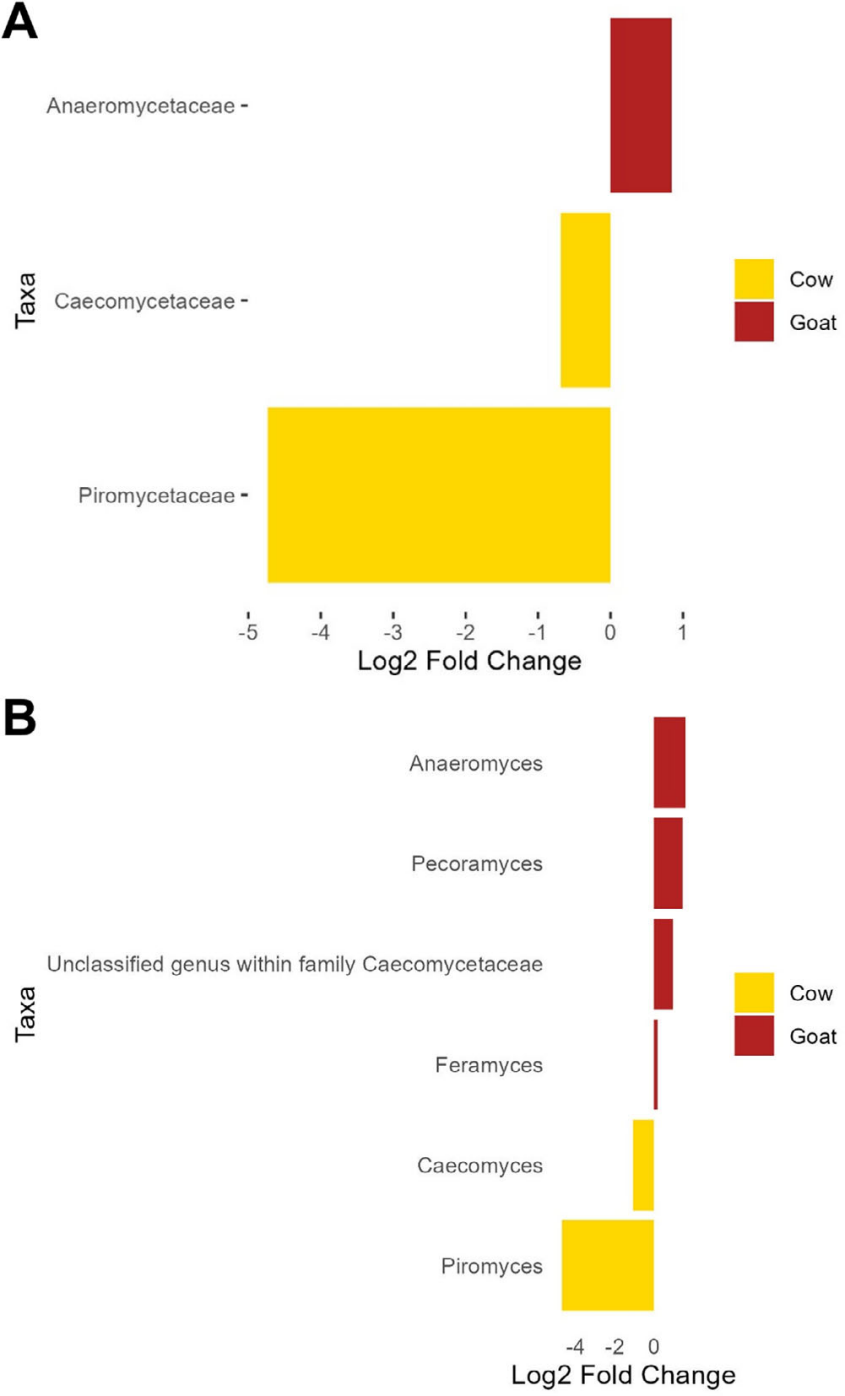
Differential abundance analysis of anaerobic fungal families (A) and genera (B) performed using DESeq2 with apeglm shrinkage. Bars display the log2 fold change (LFC) for taxa with significant differences between goats and cows (*p*adj < 0.05). Positive LFC values indicate taxa enriched in goats (red), whereas negative LFC values represent taxa enriched in cows (yellow).

**TABLE 1:** DESeq2 analysis result table for anaerobic fungi families.

Family	baseMean	log2FC	lfcSE	*p* value	*p*adj
Caecomycetaceae	13,257.16	−0.688	0.265	0.002	0.019
Piromycetaceae	245.59	−4.730	1.401	0.006	0.025
Anaeromycetaceae	6062.12	0.844	0.470	0.010	0.027
Unclassified family within order Neocallimastigales	425.04	−0.247	0.497	0.146	0.291
genera_incertae_sedis	1308.57	−0.081	0.431	0.499	0.665
Index	17.48	−0.173	0.304	0.436	0.665
Neocallimastigaceae	8917.32	0.055	0.129	0.620	0.709
Unclassified phylum within domain unassigned	937.37	0.060	0.318	0.788	0.788

Abbreviations: log2FC, log2 fold change; LfcSE, log2 fold change standard error; *p*adj, adjusted *p* value.

**TABLE 2:** DESeq2 analysis result table for anaerobic fungi genera.

Genus	baseMean	log2FC	lfcSE	*p* value	*p*adj
Caecomyces	7408.81	−1.08	0.230	7.58E‐07	1.21E‐05
Anaeromyces	5730.90	1.61	0.459	7.53E‐05	6.02E‐04
Unclassified genus within family Caecomycetaceae	1409.77	0.97	0.296	4.33E‐04	2.31E‐03
Feramyces	214.48	0.17	0.725	5.22E‐03	2.09E‐02
Pecoramyces	2229.87	1.45	0.711	6.79E‐03	2.17E‐02
Piromyces	267.71	−4.69	1.231	9.94E‐03	2.65E‐02
RH1	1072.48	−0.61	1.021	7.81E‐02	1.78E‐01
Unclassified family within order Neocallimastigales	399.60	−0.65	0.825	1.36E‐01	2.73E‐01
Cyllamyces	3551.49	−0.26	0.201	1.71E‐01	3.04E‐01
Orpinomyces	6271.13	−0.20	0.220	3.35E‐01	5.36E‐01
AL8	22.93	−0.22	0.756	4.24E‐01	6.16E‐01
Capellomyces	402.79	−0.23	0.406	5.05E‐01	6.22E‐01
SK3	499.98	0.22	0.746	4.67E‐01	6.22E‐01
Liebetanzomyces	50.90	0.12	0.719	6.80E‐01	7.49E‐01
Neocallimastix	238.06	−1.46	0.892	7.02E‐01	7.49E‐01

Abbreviations: log2FC, log2 fold change; LfcSE, log2 fold change standard error; *p*adj, adjusted *p* value.

## 4 Discussion

Ruminants provide high‐quality food for human nutrition, rich in proteins, essential amino acids, vitamins and minerals, due to their digestive system populated by a complex, diverse and active microbial community capable of fermenting fibrous plant materials otherwise indigestible for mammals (Qi et al. 2023). The ecological role of ruminants is also irreplaceable, owing to their participation in grassland management, nutrient recycling, natural fertilization and maintaining soil health, especially in cattle raised on pastures. The microbiology of the digestive tract of ruminants, especially the rumen, has, therefore, long been the subject of considerable attention in connection with the productivity and health of animals, the quality of their products intended for consumers and not least, with ecological impacts.

AF were discovered as part of the herbivore microbiome with a significant delay compared to bacteria and protozoa, but their importance for fibre digestion is unique and essential (Hess et al. 2020). Despite their recognized functional importance, these fungi remain vastly underexplored compared to bacterial communities (Hartinger and Zebeli 2021). Studies investigating fungal diversity in ruminants, especially in relation to host species, remain limited. A significant study in this regard is that of Meili et al. (2023), which highlights the significant influence of the host on the diversity of AF. Our work therefore focused on this topic and we performed an analysis of AF diversity in faeces of two ruminant species reared under the same conditions. We realize that faecal samples do not capture the same profile of AF as rumen samples and are less diverse (Meili et al. 2024). However, this approach represents a practical and non‐invasive proxy for assessing the composition of the rumen microbial community respecting animal welfare according to the 3R principles (reduce, replace, refine) by avoiding the use of cannulated animals (Rippstein et al. 2025).

Our study targeted the D2 region of the LSU rRNA gene, which is increasingly being used instead of ITS1 spacer. There are several reasons for this choice. The D2 region shows a lower degree of sequence heterogeneity, provides better distinguishing of AF genera, and eliminates the possibility of ambiguous alignments and inaccurate taxonomic classification. This region is not so AT‐rich as ITS1 and is less variable in length (Hanafy et al. 2020).

Therefore, the use of the D2 region is now recommended for rigorous studies of AF community. However, ITS1 remains useful, but its limitations due to high variability within a strain must be taken into account.

In our results, significant differences were observed in both alpha and beta diversity indices between two studied groups. In goat samples, compared to cow samples, lower faecal anaerobic fungal diversity richness and evenness were revealed. This is in contrast with the findings of Meili et al. 2023, 2024), who reported no significant differences in alpha diversity between goats and cattle. However, Meili et al. (2024) detected that goat harboured the least diverse AF community (ANOVA *p* value <0.03) when comparing families *Bovidae*, *Cervidae* and *Antilocapridae*. In the study by Meili et al. (2024), the alpha diversity was much lower for goats compared to cattle; however, the difference was not significant due to the high cattle group dispersion within the samples of cattle.

Beta diversity, assessed using the weighted UniFrac distance matrix, also revealed significant differences between the two studied groups. The PCoA plot showed clear separation between cows and goats, reflecting distinct fungal communities driven by host species differences. This is consistent with the findings of Meili et al. (2023), who reported that, regardless of the type of beta diversity indices used, animal species appear to be the most significant factor, causing 16%–21% of the variance in the anaerobic fungal community structure, indicating that host species is a key determinant of gut anaerobic fungal composition. Our results, showing statistically significant differences in diversity levels and community structure, thus support the opinion that host species play an important role in shaping the AF community.

Samples from goats showed a highly similar taxonomic composition of AF but differed in the relative abundance of species. Goats formed two subgroups, despite they were all housed on the same farm, belonged to the same breed, consumed the same diet and were kept under uniform management conditions. One subgroup was dominated by *Anaeromyces* (family Anaeromycetaceae), a genus characterized by extensive rhizoid development and strong cellulolytic activity, playing a significant role in plant fibre degradation (Hess et al. 2020; Pratt et al. 2025). The second subgroup was dominated by *Caecomyces* (family Caecomycetaceae), a genus characterized by the absence of extensive rhizoids and a lower fibre‐degrading capacity compared to other anaerobic fungal genera (Wubah 2004). This clustering pattern is unexpected under standardized conditions and suggests the influence of additional biological or ecological factors. One possibility is the role of host genetics. Although not formally tested in this study, observational records suggest that goats within each subgroup may be closely related, potentially sharing a maternal lineage. Maternal transfer of microbes begins at birth through exposure to the dam's vaginal and faecal microbiota, as well as through colostrum, skin contact and saliva (Yeoman et al. 2018; Voland et al. 2025). These early microbial communities form the foundation of the offspring's gut and faecal microbiota (Huuki et al. 2024). Another explanation could be an early‐life microbial colonization, which can have long‐lasting effects on gut community structure. Goats raised in close contact may have acquired similar initial microbial populations. In particular, the presence of adult goats as companions to newborn goat may facilitate microbial transfer, accelerating both microbial colonization and functional development of the rumen in young ruminants (Palma‐Hidalgo et al. 2021). These effects, where early microbial colonizers shape the assembly of later communities, are well documented in microbiome ecology (Guo et al. 2020; Arshad et al. 2021) and may help explain the divergence observed in our findings. Another ecological explanation may involve social behaviour and spatial foraging patterns. Although all goats were kept together, social grouping and preferential grazing behaviours could result in differential exposure to environmental microbial sources. Studies on feral goat populations have revealed that goats naturally form dynamic social structures characterized by the formation of stable subgroups or ‘cliques’ within the larger population (Stanley and Dunbar 2013; Zobel et al. 2018). Lastly, microbial interactions within the gut ecosystem may play a role. AF interact with bacteria, archaea and protozoa through competition and metabolite exchange. Differences in bacterial community composition, shaped by host and environmental factors, can indirectly influence fungal colonization and dominance through cross‐feeding networks or competitive exclusion (Culp and Goodman 2023).

Compared with published data, our results support the finding of Meili et al. (2024), who described goats‐specific pairwise AF genus‐animal species associations with *Caecomyces*, *Orpinomyces* and *Cyllamyces*. However, the association of goats with *Neocallimastix* was not observed in our study. Moreover, we did not detect any sequences belonging to NY44, NY47 and *Joblinomyces*, which were reported as significantly increased in goat samples compared to other animal species in the study by Meili et al. (2023).

The AF taxonomic composition in cow samples was more individual for each animal. Although *Caecomyces*, *Cyllamyces* and *Orpinomyces* dominated most samples, a higher percentage of *Piromyces* was found in only three samples and increased abundance of *Neocallimastix* was detected only in one sample (CN6). Among uncultured AFs, RH1 and SK3 were more abundant, whereas percentage of AL8 was very low. Enrichment of *Cyllamyces* was observed also in cattle faecal samples by both studies of Meili et al. (2023, 2024). On the other hand, significantly increased *Anaeromyces* and AL8 (Meili et al. 2023) were not found in our study.

It is important to note that relative abundance data do not directly quantify functional activity. Although some genera are widely recognized for their fibrolytic capabilities, their enrichment in one host does not necessarily imply increased cellulolytic activity. Our interpretations, therefore, reflect assumed functional tendencies on the basis of established physiological traits, rather than direct measurements of enzymatic activity or fibre degradation.

There are numerous biological and ecological differences between cows and goats which may explain their divergent fungal communities. These include differences in body size, rumen volume, rumen content passage rate, feeding behaviour, feed intake, digestive physiology, nutrient utilization and overall digestive efficiency (Silanikove 2000; Hume 2005; Giger‐Reverdin et al. 2020). Some of these traits are intrinsic, rooted in evolutionary and physiological adaptations, whereas others arise from how each species interacts with its environment and available resources. An important limitation of our study is that cows and goats differ markedly in grazing selectivity; goats are browsers with highly selective feeding habits, whereas cows are grazers that consume a more homogeneous diet (Sanon et al. 2007). These behavioural differences can lead to systematic variation in the botanical and chemical composition of ingested forage, which, in turn, may influence the fungal taxa that colonize and persist in the gastrointestinal tract. These differences likely shape the gut ecosystems in species‐specific ways which select for particular fungal assemblages. Another limitation is that animals belonging to the different host species were sampled within a single farm. Although this approach reduced the potential confounding effects associated with differences in management practices, environmental conditions, and feeding strategies among farm, it also limits the generalizability of the observed species‐related differences. Nevertheless, the use of a single farm allowed the comparison of the different species under relatively homogeneous management and environmental conditions, thereby reducing an additional source of variability that could have arisen from the inclusion of multiple farms. Future studies involving replicated herds or flocks across multiple farms, together with different dietary and management conditions, will be necessary to determine whether the observed patterns are consistent across host species and production systems and to disentangle host‐species effects from farm‐specific effects. Taken together, our findings support the growing consensus that anaerobic fungal communities are closely tied to host species (Liggenstoffer et al. 2010; Li et al. 2021). This host specificity is likely driven by a combination of genetic, physiological and ecological factors which influence microbial colonization, retention and function within the gastrointestinal tract.

## 5 Conclusion

This study demonstrates that host species significantly influence the diversity and composition of faecal anaerobic fungal communities in ruminants, even when animals are maintained under similar feeding management. Cows exhibited higher anaerobic fungal richness and evenness than goats. In contrast, goats formed two distinct subgroups with highly similar AF community structure. Differential abundance analysis confirmed host‐specific patterns.

## Author Contributions

Conceptualization: Kateřina Olša Fliegerová, Giuseppe Moniello and Silvana Mattiello. Methodology: Kateřina Olša Fliegerová, Jakub Mrázek, Radka Roubalová, Janet Luthar and Tiziana Maria Mahayri. Formal analysis and investigation: Alessia Serra, Janet Luthar, Stefania Celozzi and Tiziana Maria Mahayri. Resources: Silvana Mattiello and Stefania Celozzi. Data curation: Tiziana Maria Mahayri. Writing – original draft preparation: Tiziana Maria Mahayri. Writing – review and editing: Kateřina Olša Fliegerová and Giuseppe Moniello.

## Funding

This research was carried with the contribution of funds obtained from Fondazione di Sardegna—Italy, FDS2223MONIELLO—CUP J83C22000160007 (responsible Prof. G. Moniello) and of Fondo di Ateneo per la Ricerca 2020, University of Sassari (recipient G. Moniello).

## Ethics Statement

The animals were treated humanely in accordance with the European Union (EU) legislation Directive 2010/63/EU on the protection of animals use d for experimental and other scientific purposes. Experimental procedure and animal handling were performed according to arrive. Prior to conducing the experiment an informed consent was obtained from the farmer owner.

## Conflicts of Interest

The authors declare no conflicts of interest.

## Supporting information




**Supporting File**: vms371291‐sup‐0001‐SuppMat.docx.

## Data Availability

The data that support the findings of this study are openly available in SRA at https://www.ncbi.nlm.nih.gov/sra/?term=PRJNA1268001, reference number PRJNA1268001.
