# Supplementary material for: Comparison of Faecal Anaerobic Fungi of Cows and Goats Offered Similar Diet
Source: Vet Med Sci. 2026 Oct 30;12(6):e71291. doi: 10.1002/vms3.71291 (PMC13720522; doi:10.1002/vms3.71291)
Supplement: Supplementary file 1 [file VMS3-12-e71291-s001.docx]

**Supplementary Material**

**Comparison of fecal anaerobic fungi of cows and goats offered similar diet**

Tiziana Maria Mahayri ^1,2^, Alessia Serra ^2^, Janet Luthar ^3^, Radka Roubalová ^3^, Giuseppe Moniello ^2^, Silvana Mattiello ^4^, Stefania Celozzi ^4^, Jakub Mrázek ^1^, Kateřina Olša Fliegerová ^1^

^1^	Laboratory of Anaerobic Microbiology, Institute of Animal Physiology and Genetics, Czech Academy of Science, 14220 Prague, Czech Republic

^2^	Department of Veterinary Medicine, University of Sassari, 07100 Sassari, Italy

^3^	Laboratory of Cellular and Molecular Immunology, Institute of Microbiology of the Czech Academy of Sciences, Prague, Czech Republic

^4^	Department of Agricultural and Environmental Sciences—Production, Landscape, Agroenergy, University of Milan, 20133 Milan, Italy

**Table S1.** Animals’ characteristics

| Sample name | Animal species | Age | Lactating | Pregnant |
| --- | --- | --- | --- | --- |
| GN 4 | Goat | 6 years | Yes | No |
| GN 6 | Goat | 4 years | Yes | No |
| GN 7 | Goat | 3 years | No | Yes |
| GN 14 | Goat | 3 years | No | Yes |
| GN 15 | Goat | 3 years | No | Yes |
| GN 16 | Goat | 4 years | No | Yes |
| GN 17 | Goat | 5 years | No | Yes |
| GN 21 | Goat | 3 years | No | Yes |
| GN 22 | Goat | 7 years | No | Yes |
| GN 24 | Goat | 7 years | No | Yes |
| GN 25 | Goat | 2 years | No | Yes |
| GN 27 | Goat | 3 years | No | Yes |
| GN 28 | Goat | 7 years | No | Yes |
| GN 29 | Goat | 4 years | No | Yes |
| GN 30 | Goat | 6 years | No | Yes |
| CN 1 | Cow | 3 years | Yes | Yes |
| CN 2 | Cow | 3 years | Yes | Yes |
| CN 6 | Cow | 7 years | Yes | Yes |
| CN 9 | Cow | 7 years | Yes | Yes |
| CN 10 | Cow | 3 years | Yes | Yes |
| CN 15 | Cow | 4 years | No | Yes |
| CN 16 | Cow | 5 years | No | Yes |
| CN 17 | Cow | 8 years | No | Yes |
| CN 19 | Cow | 7 years | No | Yes |
| CN 20 | Cow | 7 years | No | Yes |
| CN 21 | Cow | 7 years | No | Yes |
| CN 22 | Cow | 7 years | No | Yes |
| CN 23 | Cow | 7 years | No | Yes |
| CN 24 | Cow | 8 years | No | Yes |
| CN 26 | Cow | 5 years | No | Yes |
| CN 28 | Cow | 9 years | No | Yes |

**Table S2.** Summary of the number of raw reads and filtered high-quality reads.

| Sample Id | input | filtered | percentage of input passed filter | denoised | merged | percentage of input merged | non-chimeric | percentage of input non-chimeric |
| --- | --- | --- | --- | --- | --- | --- | --- | --- |
| GN 4 | 47716 | 41509 | 86.99 | 41453 | 39962 | 83.75 | 34653 | 72.62 |
| GN 6 | 71432 | 65043 | 91.06 | 64909 | 61596 | 86.23 | 50504 | 70.7 |
| GN 7 | 65542 | 59605 | 90.94 | 59403 | 55987 | 85.42 | 46164 | 70.43 |
| GN 14 | 67535 | 61466 | 91.01 | 61316 | 58355 | 86.41 | 47272 | 70 |
| GN 15 | 57454 | 49856 | 86.78 | 49784 | 47376 | 82.46 | 39691 | 69.08 |
| GN 16 | 53913 | 47339 | 87.81 | 47263 | 46040 | 85.4 | 40566 | 75.24 |
| GN 17 | 56713 | 50453 | 88.96 | 50326 | 47289 | 83.38 | 39169 | 69.07 |
| GN 21 | 52189 | 47196 | 90.43 | 47135 | 45959 | 88.06 | 44477 | 85.22 |
| GN 22 | 68355 | 61458 | 89.91 | 61389 | 59227 | 86.65 | 55880 | 81.75 |
| GN 24 | 60947 | 53043 | 87.03 | 52986 | 50782 | 83.32 | 48208 | 79.1 |
| GN 25 | 53885 | 48664 | 90.31 | 48589 | 46600 | 86.48 | 43630 | 80.97 |
| GN 27 | 61675 | 55519 | 90.02 | 55454 | 53640 | 86.97 | 51126 | 82.9 |
| GN 28 | 56058 | 50119 | 89.41 | 50008 | 48201 | 85.98 | 45336 | 80.87 |
| GN 29 | 55988 | 50573 | 90.33 | 50475 | 48694 | 86.97 | 46525 | 83.1 |
| GN 30 | 49554 | 43360 | 87.5 | 43309 | 42274 | 85.31 | 41066 | 82.87 |
| CN 1 | 59364 | 52294 | 88.09 | 51855 | 44734 | 75.36 | 31358 | 52.82 |
| CN 2 | 54222 | 48770 | 89.95 | 48461 | 41892 | 77.26 | 23828 | 43.95 |
| CN 6 | 56223 | 50879 | 90.49 | 50629 | 46640 | 82.96 | 35485 | 63.11 |
| CN 9 | 41658 | 37337 | 89.63 | 37100 | 32384 | 77.74 | 23106 | 55.47 |
| CN 10 | 53680 | 49024 | 91.33 | 48703 | 43252 | 80.57 | 29871 | 55.65 |
| CN 15 | 47842 | 43412 | 90.74 | 43023 | 36093 | 75.44 | 22749 | 47.55 |
| CN 16 | 53826 | 48667 | 90.42 | 48280 | 41353 | 76.83 | 27907 | 51.85 |
| CN 17 | 34681 | 30057 | 86.67 | 29887 | 25602 | 73.82 | 19340 | 55.77 |
| CN 19 | 53753 | 48310 | 89.87 | 47925 | 40317 | 75 | 26508 | 49.31 |
| CN 20 | 48743 | 42140 | 86.45 | 41893 | 35846 | 73.54 | 26846 | 55.08 |
| CN 21 | 38492 | 33890 | 88.04 | 33596 | 29268 | 76.04 | 21810 | 56.66 |
| CN 22 | 47802 | 43385 | 90.76 | 43053 | 35611 | 74.5 | 23809 | 49.81 |
| CN 23 | 37367 | 33957 | 90.87 | 33628 | 27731 | 74.21 | 18744 | 50.16 |
| CN 24 | 63974 | 57829 | 90.39 | 57509 | 51698 | 80.81 | 41125 | 64.28 |
| CN 26 | 64130 | 57894 | 90.28 | 57505 | 51461 | 80.24 | 40005 | 62.38 |
| CN 28 | 43779 | 37910 | 86.59 | 37762 | 34422 | 78.63 | 28571 | 65.26 |
| TOTAL | 1,678,492 | 1,500,958 | 89.42 | 1,494,608 | 1,370,286 | 81.64 | 1,115,329 | 66.45 |

**Table S3.** Summary of alpha diversity indices of fungal community in cows and goats (means ± SD).

| Index | Cows | Goats | *p-value* |
| --- | --- | --- | --- |
| Chao1 | 98.33 ± 16.54 | 46.22 ± 5.53 | 0.000002* |
| Pielou’s Evenness | 0.81 ± 0.06 | 0.77 ± 0.05 | 0.04* |
| Shannon | 5.32 ± 0.48 | 4.26 ± 0.19 | 0.000002* |
| Faith’s pd | 1.87 ± 0.19 | 0.91 ± 0.08 | 0.000002* |

* Significant difference (p < 0.05)

**Table S4.** Relative abundance (means ± SD) of bacterial taxa at family and genus levels in the feces of goats and cows.

| Taxonomic levels | Cow | Goat |
| --- | --- | --- |
| Family |  |  |
| Anaeromycetaceae | 6.75 ± 3.50 | 30.40 ± 15.78 |
| Caecomycetaceae | 37.79 ± 9.80 | 41.39 ± 20.38 |
| Neocallimastigaceae | 33.20 ± 13.79 | 26.10 ± 4.24 |
| Piromycetaceae | 6.03 ± 12.39 | 0.03 ± 0.04 |
| Unassigned | 5.07 ± 1.88 | 1.44 ± 1.28 |
| Genera incertae sedis | 11.16 ± 6.3 | 0.64 ± 0.66 |
| Genus |  |  |
| *AL8* | 0.13 ± 0.15 | 0.01 ± 0.04 |
| *Anaeromyces* | 4.87 ± 2.70 | 29.41 ± 16.26 |
| *Caecomyces* | 21.73 ± 7.43 | 25.85 ± 18.28 |
| *Capellomyces* | 1.66 ± 0.99 | 0.91 ± 0.61 |
| *Cyllamyces* | 12.54 ± 5.50 | 10.54 ± 5.42 |
| *Feramyces* | 1.95 ± 2.94 | 0.00 ± 0.00 |
| *Khyollomyces* | 0.00 ± 0.00 | 0.00 ± 0.00 |
| *Liebetanzomyces* | 0.23 ± 0.40 | 0.08 ± 0.10 |
| *Neocallimastix* | 5.06 ± 9.59 | 0.24 ± 0.16 |
| *Orpinomyces* | 24.42 ± 11.47 | 14.47 ± 3.09 |
| *Pecoramyces* | 1.77 ± 1.51 | 11.39 ± 7.11 |
| *Piromyces* | 6.03 ± 12.39 | 0.03 ± 0.04 |
| *RH1* | 5.97 ± 5.59 | 0.01 ± 0.01 |
| *SK3* | 3.27 ± 3.18 | 0.00 ± 0.00 |
| *Unclassified family within order Neocallimastigales* | 1.79 ± 1.52 | 0.63 ± 0.62 |
| *Unclassified genus within family Caecomycetaceae* | 3.52 ± 1.52 | 4.99 ± 3.32 |
| Unassigned | 5.07 ± 1.88 | 1.44 ± 1.28 |

**Figure S1.** Rarefaction curves representing the sequencing depth (number of reads) and the number of ASVs (sequence variants) found in feces of goats and cows.
